# Advances in the *in Vivo* Raman Spectroscopy of Malignant Skin Tumors Using Portable Instrumentation

**DOI:** 10.3390/ijms160714554

**Published:** 2015-06-26

**Authors:** Nikolaos Kourkoumelis, Ioannis Balatsoukas, Violetta Moulia, Aspasia Elka, Georgios Gaitanis, Ioannis D. Bassukas

**Affiliations:** 1 Department of Medical Physics, School of Health Sciences, University of Ioannina, 45110 Ioannina, Greece; E-Mails: ibalats@cc.uoi.gr (I.B.); biomoulia@yahoo.gr (V.M.); e.aspa@yahoo.com (A.E.); 2 Department of Skin and Venereal Diseases, School of Health Sciences, University of Ioannina, 45110 Ioannina, Greece; E-Mails: ggaitan@uoi.gr (G.G.); ibassuka@cc.uoi.gr (I.D.B.)

**Keywords:** Raman spectroscopy, *in vivo* spectroscopy, skin cancer

## Abstract

Raman spectroscopy has emerged as a promising tool for real-time clinical diagnosis of malignant skin tumors offering a number of potential advantages: it is non-intrusive, it requires no sample preparation, and it features high chemical specificity with minimal water interference. However, *in vivo* tissue evaluation and accurate histopathological classification remain a challenging task for the successful transition from laboratory prototypes to clinical devices. In the literature, there are numerous reports on the applications of Raman spectroscopy to biomedical research and cancer diagnostics. Nevertheless, cases where real-time, portable instrumentations have been employed for the *in vivo* evaluation of skin lesions are scarce, despite their advantages in use as medical devices in the clinical setting. This paper reviews the advances in real-time Raman spectroscopy for the *in vivo* characterization of common skin lesions. The translational momentum of Raman spectroscopy towards the clinical practice is revealed by (i) assembling the technical specifications of portable systems and (ii) analyzing the spectral characteristics of *in vivo* measurements.

## 1. Introduction

The spectrum of skin cancers in humans currently encompasses the most frequent neoplasm types by tissue of origin and the most costly cancer categories to treat [1]. From the different types of skin neoplasms, cancers of keratinocytic origin (epithelial skin cancer, formerly designated preferentially as non-melanoma skin cancer (NMSC)) form the most frequent category. The two most common skin cancer types of this group, basal cell carcinoma (BCC) and squamous cell carcinoma (SCC) comprise ~95% of all skin cancers and together with the third most frequent, malignant melanoma (MM), constitute 99% of the incidence of all skin neoplasms. Several studies have documented the increasing trends in skin cancer occurrence all over the world [2,3,4,5,6,7]. Furthermore, distinct subpopulations exist with significantly increased risk for developing tumors in this organ like patients with multiple nevi [8] and those that are on long-term iatrogenic immunosuppresion [9]. However, the aforementioned three most frequent skin cancer types, *i.e.*, BCC, SCC and MM, differ substantially in their biological aggressiveness and relevant prognosis. Keratinocytic skin cancers are typically curable, especially if diagnosed early enough [10] and despite the increasing incidence, their mortality rates remain low [11,12]. Notably for BCC, there are reports of rapidly declining mortality rates despite the marked incidence increase during the same period [13]. On the other hand, MM is a biologically aggressive neoplasm. The diagnosis is often ambiguous and the course of the disease strongly depends on the tumor stage [14]. In general, it is highly unpredictable at the individual patient’s level; the prognosis is markedly worse in advanced stages with the five-year survival rate as low as 16%. However, when melanoma is detected and excised in the initial *in situ* stage, survival reaches 99% and the disease can be considered practically curable [15].

Early diagnosis is critical for the successful treatment of skin neoplasms. The initial step of clinical evaluation in which suspicious lesions are selected for targeted invasive assessment with biopsies is crucial because the selection process is challenging even for the experienced clinician. Few lesions have to be picked up for biopsy between a multitude of benign and malignant and look-alike skin alterations coexist side by side in skin areas severely damaged by excessive life-long sunlight exposure. The specificity and the sensitivity of the above clinical procedure vary from 40% to 80%, imposing substantial uncertainty on this diagnostic process [16]. Moreover, it is very important to limit the use of skin biopsies since this invasive and time-consuming process is often associated with substantial patient discomfort. At this point, unbiased clinical decisions based on non-invasive, real-time and time-saving diagnostic techniques are preferable to facilitate the management of the increasing number of patients with skin cancer [17,18]. From the many optical spectroscopy techniques under evaluation for the early, non-invasive detection of skin lesions, two vibrational spectroscopy modalities seem to be the most promising: Fourier Transform Infrared (FTIR) and Raman Spectroscopy [16,19,20]. The strong absorption of mid-infrared radiation by water molecules limits the clinical *in vivo* applicability of the FTIR technique [21], although a lot of progress has been achieved towards this issue [22]. On the other hand, Raman spectroscopy is able to detect spectroscopic fingerprints of tissues in their native state within clinically acceptable measurement time (seconds) without the need for tissue pre-processing, like staining or fixation.

Raman spectroscopy is based on the inelastic scattering of photons exchanging energy via molecular vibrational modes. Due to the fact that molecular energy levels are quantized and unique for each molecule, Raman spectra feature discrete and chemical bonds-specific bands, providing a “molecular fingerprint” of the samples under study [23]. Thus, Raman active molecules (*i.e.*, with anisotropic polarizability) can give spectroscopic signals with significant information about the chemical composition of the sample. The technique has been applied in a variety of biological and medical research branches as a reliable modality for the *in situ* diagnosis of malignancy at tissue level or for the analysis of subcellular molecular composition of tissues [24]. Moreover, it can be employed to determine steps in tumor progression or to evaluate response to radiation therapy [25].

This paper reviews the advances in the use of portable Raman systems for the clinical, *in vivo* spectroscopic characterization of skin lesions in real-time with emphasis in the diagnosis and management of the three most common skin cancers (BCC, SCC and MM). Where possible, we compare the experimental outcomes in an effort to identify the best practices for the clinical environment.

## 2. Instrumentation and Experimental Considerations

### 2.1. Portable Raman Acquisition Systems

Raman spectroscopy is an optical spectroscopic technique based on the inelastic scattering of monochromatic light. Raman scattering involves inelastic collisions between the photons of an irradiating laser beam and the sample (or tissue) molecules. The absorption of photons, results in energy exchange between photons and tissue molecules generating vibrations within the material that are molecule specific. A nonlinear polyatomic molecule with N atoms has 3*N*-6 modes of vibration, known as “normal modes” which are related to a fundamental frequency of vibration and symmetry. When photons are reemitted, their energy, and hence frequency, is shifted in comparison to the excitation frequency, yielding the so-called “Raman shift”. The Raman shift is independent of the excitation (incident) wavelength, which means that it is constant and unique for the different molecules. Thus, the evaluation of the scattered light is transformed to the vibrational Raman spectrum of the sample, which contains substantial qualitative and quantitative information about the chemical composition of the examined probes. Since the detection of certain Raman bands correlates with the presence of specific molecules in a probe, Raman spectra can be used to differentiate between tissues of different pathological conditions. Raman cross section is wavelength dependent and varies according to 1/λ^4^, where λ is the excitation wavelength [26]. Thus, the intensity of the Raman peaks is proportional to f^4^ (where f is the laser frequency) and, for example, a 488 nm laser gives an almost seven times more intense Raman signal than a 785 nm laser. Moreover, the transmission efficiency of the optics and detector sensitivity is also wavelength dependent [27]. The excitation wavelength is a significant parameter for the analysis of skin lesions because it has a major impact on the scattered intensity, the auto-fluorescence and the signal attenuation. Skin research can be conducted in a wide range of excitation frequencies from visible to near infrared (NIR) wavelengths and this has been an obstacle to compare methodologies and results. NIR generate lower auto-fluorescence intensities compared to the excitation with visible light but at the same time poorer scattering intensity too, resulting into lower sensitivity and thus to a lower signal to noise ratio. Laser sources at 785 or 830 nm are preferred for clinical Raman applications because the operation at these wavelengths combines reduced auto-fluorescence and adequate tissue penetration depth [28]. Sources at 1064 nm also have a favorable profile of signal to noise ratio for skin applications but they usually require increased irradiation doses and annoying prolonged spectra acquisition times [29,30]. Nevertheless, optimally employed FT-Raman spectrometers at this excitation wavelength demonstrate relatively high signal to noise ratio [31]. The light attenuation in the skin tissue was evaluated on excised human skin by confocal Raman microspectroscopy [32,33,34,35] and confirmed that Raman signals are suitable for skin measurements at various depths (up to a few hundred micrometers) depending on the spectrometer setup. The safety issues of tissue irradiation and exposure times are regulated by consensus directives released by international organizations (ANSI Z136, ICNIRP, and IEC 60825-1).

Table 1 summarizes the major operation parameters (wavelength, power, focal spot size, and signal integration time) employed in *in vivo* studies of a variety of skin lesions with the use of portable Raman spectroscopy devices for the acquisition of spectra in real-time (the blue line separates *in vivo* studies which did not aim to the classification of common skin lesions but solely to biochemical characterization). In the clinical setting with the appropriate equipment, Raman spectroscopy does not require any specific tissue pre-treatment except from possible superficial cleaning of excess sebum with ethanol [16]. *Ex vivo* studies of skin tissue material samples and biopsy probes are not included in Table 1, even if they have been carried out with portable setups.

The diverse Raman implementations presented in Table 1 imply that there is neither any optimal experimental design nor any standardization among setups. The data clearly indicate that it is difficult to quantify the tradeoff between excitation wavelength and lower scattering cross section with auto-fluorescence intensity since the detection of a specific Raman band depends on both the experimental parameters and the (heterogeneous) composition of skin tissue. Tfayli *et al.* [28] have studied the wavelength effect on pig skin epidermis by recording spectra at 532, 633, and 785 nm and analyzing the variability and the repeatability of the experiments along with the effect of exposure time and in-depth signal attenuation. They suggested that the excitation wavelength at 785 nm is advantageous (compared to 532 and 633 nm) for the fingerprint skin region, having lower signal attenuation. Yet, in principle, it is rather difficult to extrapolate outcomes and directly compare results by different research groups using different wavelengths and instrumentation. Varying sampling rates and different statistical classification schemes add another level of complexity for the preparation of an accepted clinical protocol. Calibration of portable Raman systems with standard samples of exact composition, resembling human skin, is possibly a requirement for the future.

**Table 1:** Technical details of clinical applications of portable Raman spectroscopy in skin cancer diagnosis.

Cancer Type	Technique	Raman Excitation Wavelength (nm)	Spot Size (mm)	Power (mW)	Signal Integration Time (s)	Number of Skin Lesions Studied and/or Patients	Reference
MM, BCC, SCC, actinic keratosis (AK), atypical nevi, melanocytic nevi, blue nevi, and seborrheic keratoses	Raman	785	3.5	300	1	518 (453 patients)	[36]
BCC, inflammatory scar tissues	Raman + OCT ^a^	785	0.044	40	30	1 patient	[15]
MM, BCC, SCC, pigmented nevi	Raman	785	1	150	30	50	[37]
MM, BCC, SCC, pigmented nevi	Raman + OCT	785	1	150	30	23, 50	[38,39]
MM, BCC, SCC, pigmented nevi	Raman	785	0.1	17	10	137	[40,41]
BCC, SCC, inflammatory scar tissues	Raman	825	0.005 ^b^	40	30	21 (19 patients)	[42]
BCC	Raman	830	1.6	110	30	10 patients	[43]
BCC, SCC	Raman	830	-	200	20 (2 s × 10 spectra)	31 (17 patients)	[44]
BCC, SCC, AK	Raman	830	0.17	200	20 (2 s × 10 spectra)	49 (25 patients)	[45]
MM, BCC, SCC, AK, and non-melanoma pigmented lesions	Raman	830	0.2	100	1	137 (76 patients)	[46,47]
BCC	Multi modal ^c^	830	0.2	56	4	1 (healthy) ^d^	[48]
MM, eczema, psoriatic skin, malignant Kaposi sarcomas	Raman	1064	10	-	-	1 (healthy) ^d^	[31]
MM, BCC, pigmented nevi	Raman	1064	0.1	120	480	81 (72 patients)	[49]
Carotenoid concentration in BCC and actinic keratoses	Raman	488	2	10	20	14 patients	[50]
MM	Multi modal ^e^	1064	0.08	-	35	Mice injected with human MM cells	[51]

^a^ OCT = Optical Coherence Tomography; ^b^ Value from identical instrumentation in [52]; ^c^ Raman, fluorescence, and reflectance spectroscopy; ^d^ compared with skin lesions from *in vitro* studies; ^e^ acoustic microscopy, infrared reflectance and Raman spectroscopy (proof-of-concept study); BCC: basal cell carcinoma; SCC: squamous cell carcinoma; MM: malignant melanoma.

### 2.2. Comparison between Portable and Benchtop Systems

As a rule of the thumb, the spectral quality of portable spectrometers is lower than benchtop ones due to the poorer sensitivity and scanning range [53]. However, the superior characteristics of the benchtop spectrometers (numerical aperture, lens, and focal length) and of the laser beam (intensity profile) are compensated by mass and size restrictions in the clinical environment along with the convenient accessibility of the skin lesions on the patients’ bodies via optical fibers. In most cases, bands assignment and qualitative characterization was aided by measurements performed in biopsies and no significant alterations in bands shape and positions has been detected between *in vivo* and *in vitro* experiments. Even so, the overall spectra precision was characterized as low [54] due to the low signal to noise ratio. Thus, portable Raman setups should be able to address the question whether or not a band having 2–3 times greater intensity than the noise (which is typically defined as the square root of the raw intensity of measurement) can be detected at a certain position [27]. Raman signal is effectively the sum of two parts: the raw signal attributed to the sample’s concentration of Raman active compounds and the overall noise due to (i) fluorescence (background) and (ii) the contribution from the optical components. In addition, the resulted spectra are affected by ambient light, beam powder density, measurement time and matrix effects. Biomolecules in skin tissues are by default weak Raman scatterers imposing an additional difficulty to the challenging task of skin tumors classification. In the majority of the relevant literature, the application of chemometrics was necessary to detect the subtle changes attributed to the pathological skin lesions.

## 3. Results and Discussion

### 3.1. Bands Assignment

The positions (typically reported in wavenumbers) and relative intensities of Raman bands are the major spectral characteristics for delineating the molecular background of skin diseases. Nonetheless, for optimizing the extraction of diagnostic information, three interwoven but distinct levels of spectral analysis must be combined [55]: (i) chemical analysis which pertains to the qualitative and quantitative biochemical composition of the tissue; (ii) statistical processing of the spectral variations; and (iii) integration of core spectroscopic findings with relevant clinical information. The most common Raman bands associated with pathological skin lesions together with their chemical assignment are shown in Table 2.

**Table 2:** Tentative assignment of the most prominent Raman bands showing differentiation in spectra between normal and skin cancer tissues (MM, BCC, and SCC specific).

Cancer Type	Peak Position (cm^−1^) ^a^	Assignment	Reference
BCC	500–600	S-S disulfide stretching	[56]
BCC	727	v(CN)Adenine, Lipids	[56]
BCC	746	Thymine	[56]
BCC	786–788	Nucleic acid backbone (PO_2_ symmetric stretching)	[56,57]
BCC, SCC, MM	832	Proline, hydroxyproline, tyrosine, stretch of nucleic acids, DNA (PO_2_ symmetric stretching)	[44,46,56]
BCC, SCC	920–943	v(CC) skeletal of collagen backbone Proline, hydroxyproline	[31,42,44,58]
BCC, SCC	1000–1010	Phenylalanine (ring breathing); keratin	[42,44,56]
BCC, SCC	1085–1098	v(CC) lipids Nucleic acid backbone v(PO_2_) symmetric stretching	[15,31,56,57,59]
BCC, SCC	1127–1130	Lipids v(CC) symmetric stretching of acyl-backbone, trans conformation	[44,56]
BCC, SCC	1207–1209	Tyrosine, phenylalanine	[44,56]
MM, BCC	1220–1280	Amide III (δ(NH) bending and ν(CΝ) stretching vibrations) (protein band), tropocollagen (proline-rich), v(CH) ethylene (triolein and phospholipids)	[15,31,42,43,45,46,49,56,58–61]
MM, BCC	1300	δ(CH_2_) twist, lipids	[46,60,62]
BCC	1336	CH deformations, adenine, phenylalanine	[37,38,56]
MM, BCC	1440–1460	δ(CH_2_) scissoring in lipids and δ(CH_2_) scissoring vibration in proteins	[15,42,46,56,59,62]
MM, BCC	1520–1570	Nucleic acids	[37]
MM, BCC	1640–1685	Amide I (C=O stretching), collagen, elastin	[37,38,42,43,46,59,60]
BCC	1651	Lipids (C=C stretching), phenylalanine	[56]
MM, BCC	3250	H_2_O	[49]

^a^ Approximate values.

Data in Table 2 indicate that quantitative modulations of amide moieties bands III and I are characteristic for tumor cell proliferation, probably as a result of Raman active modifications in the molecular composition of the proteins in malignant skin tissues. In particular, the majority of the studies report a significant decrease of the amide III band intensity, which is regarded to reflect important alterations in the secondary structure of the tissue proteins. It is important to note that this band gives quite strong signal with Raman but rather weak with IR spectroscopy [63]. Apart from reduced intensity, this band seems to also be shifted to higher frequencies in the case of BCC and MM [59]. Raman spectra from malignant skin lesions show lower amide I band intensities although the corresponding results are possibly tumor type specific. Significant changes are also observed in the lipids concentration. Moreover, it is expected that the concentration of nucleic acids increases in malignant tissues due to the unrestrained cell proliferation that leads to spatial overcrowding of nucleated cells [31,64] and to intensity increase of the corresponding Raman bands. Water is another major tissue constituent. Highly significant alterations in the respective water content were observed for MM and BCC [49]. Spectral fingerprints of stabilizing disulfide bridges between cysteine residues in macromolecules are localized in the lower spectra section; detection of decreased intensity of the respective bands in tissues has been attributed to protein oxidation [56]. Carotenoids play a significant role as major anti-oxidants in the skin and they possibly contribute to the mechanisms of skin cancer prevention. Hata *et al.* [50] studied the concentration of carotenoids in the skin by analyzing the intensity of the 1524 cm^−1^ peak (v(C=C) stretch vibration of the backbone). They suggested that BCC can be differentiated from healthy skin areas as a result of their significantly lower carotenoid concentration but at the same time they concluded that further analysis on the possible correlation of the dermal carotenoids with cutaneous pathology is needed. Indeed, recent studies using resonance Raman spectroscopy [65] and reflection spectroscopy [66] showed that carotenoids concentration mostly reflects nutritional habits and stress conditions. Poor nutrition, illness, and smoking are generally related to low carotenoid concentration while stress factors (solar irradiation, environmental hazards, fatigue, illness, *etc.*) contribute to the fast degradation of dermal carotenoids. Therefore, the low carotenoids concentration is undoubtedly a significant finding but probably not a biomarker.

It is evident that certain biochemical changes associated with skin malignancies can be successfully identified by Raman spectroscopy. To reduce the high number of parameters needed to characterize the variance in the acquired spectral datasets, researchers typically utilize multivariate statistical methods to generate linear discriminant models of classification [67]. In the subsequent section we also refer to the sensitivity and specificity of these models for BCC, SCC and MM.

### 3.2. Studies of NMSC and MM with Portable Raman Instrumentation

**Figure 1:**
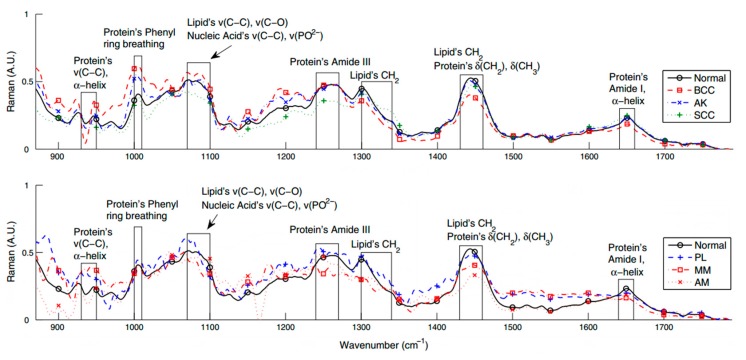
Mean Raman spectra acquired *in vivo* for basal cell carcinoma (BCC), squamous cell carcinoma (SCC), actinic keratosis (AK), nonmelanoma pigmented lesions (PL), amelanotic melanoma (AM) and normal skin. Adapted and reproduced with permission from [46].

Liu *et al.* [36] performed the most comprehensive *in vivo* study to date presenting data from Raman spectroscopic analysis of 453 patients with benign and malignant skin lesions (MM, BCC, SCC, and actinic keratoses). They concluded that distinctive Raman spectral peaks or bands cannot be uniquely assigned to any of the different skin cancer types. Moreover, they showed that the chemical fingerprints of different skin lesions are not site-dependent unlike previous findings with healthy human skin [68]. Raman spectral bands with diagnostic impact were recognized at 855, 936, 1002, 1271, 1302, 1445, 1655, and 1745 cm^−1^. It was suggested that bands in the region of 1055–1800 cm^−1^ are critical for evaluating MM. Multivariate statistical methods were necessary to evaluate information from multiple band positions determinations in order to optimize the clinical classification of skin lesions. Although the sensitivity of the above approach for the discrimination between malignant and benign skin conditions is 99%, the specificity is limited to only 15% for the differentiation between MM and benign lesions and to 17% for that of keratinocytic skin neoplasms (BCC, SCC and actinic keratoses) *vs.* benign lesions. Lim *et al.* [46] have noticed a noteworthy low intensity of the amide band I region coupled with an increase in the intensity of the lipid bands at 1310–1340 cm^−1^ in the case of MM. The decrease in the amide I band was linked to collagen degradation or to the increased melanin concentration and pigmentation of the neoplasms. Moreover, only the MM spectra exhibited peaks between 800 and 900 cm^−1^ while both BCC and MM showed peaks with lower intensity in the 1450 cm^−1^ region. The integration of spectral information in the fingerprint region (900–1800 cm^−1^) was suggested for the discrimination of skin cancers from benign lesions (Figure 1).

**Figure 2:**
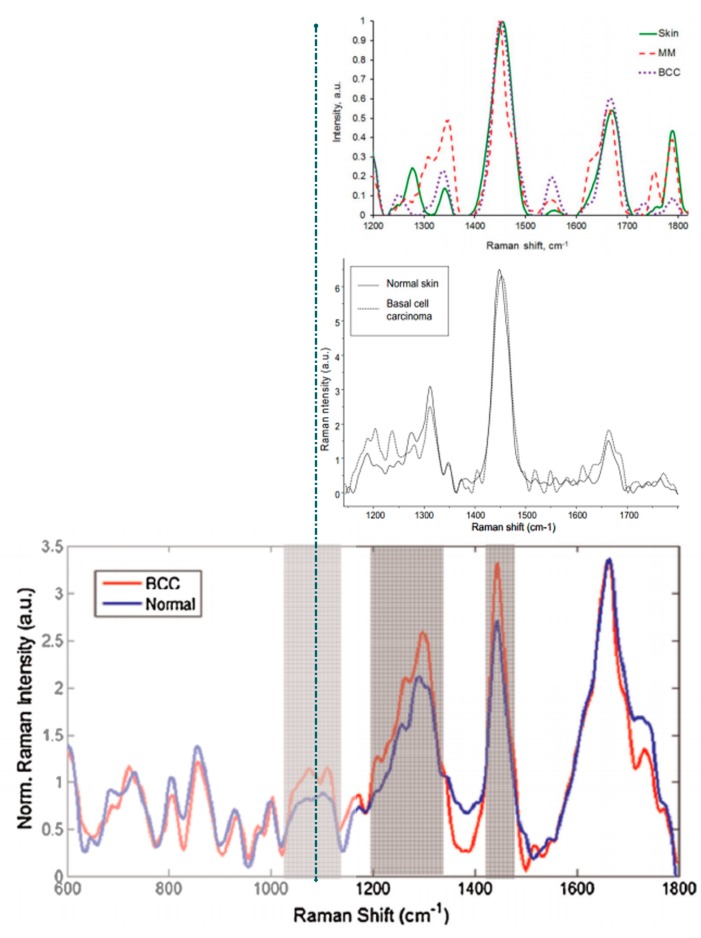
Comparative results (from three research groups) of *in vivo* Raman spectra for BCC and normal skin. Adapted and reproduced with permission from [15,37,43].

The overall lower spectral intensity of malignant skin conditions was also confirmed by other studies [69], which consistently found that BCC and SCC exhibit weaker scattering than healthy skin, probably as a result of enhanced collagen breakdown. Principal component analysis (PCA) was employed for the classification of the different lesions. With this approach the authors demonstrated a 100% sensitivity and specificity in the discrimination of MM from non-melanoma pigmented skin lesions. With the same approach SCC, BCC and actinic keratoses could also be differentiated from normal skin with sensitivity and specificity of 90% and 85%, respectively. Patil *et al.* [15] identified distinct differences between the healthy skin and BCC in the Raman bands at 1090, 1300, and 1440 cm^−1^ while Tfayli *et al.* [43] for the same kind of comparison (normal skin *vs.* BCC) performed hierarchical cluster analysis concentrating on the amide III and the CH deformation band (1410 cm^−1^) as the most promising bands; they noticed frequency shifting of the 1410 cm^−1^ peak, a weak shoulder at 1685 cm^−1^ in the BCC spectra (amide I region), differences in the amide III band and in the phospholipid content. Zakharov *et al.* [37] investigated major spectral differences between normal skin and malignant tumors (MM and BCC) using a two-step method of tumor diagnosis to optimize the sensitivity and specificity of the discrimination. The method utilized characteristic spectral intensities in the 1300–1340, 1440–1460, and 1640–1680 cm^−1^ ranges. Plots of intensities’ relative difference were constructed for healthy and malignant tissues; discriminant analysis and support vector machines assigned tissue classes with 88.9% sensitivity and 87.8% specificity for the *in vivo* Raman measurements. The authors reported a significant improvement in MM identification using this two-step classification. The same research group enhanced their technique by combining Raman spectroscopy with OCT [38] and in a recent publication, combined OCT, backscattering (BS) and Raman spectroscopy [39]. OCT is a highly accurate method for BCC diagnosis while BS was utilized for the qualification of tumor boundaries. This combined device coupled with the two-step analysis showed an increase accuracy of diagnosis by 9% for sensitivity and 8% for specificity compared to the values obtained by each method separately. Comparative results for BCC *vs.* normal tissues are depicted in Figure 2. Common significant spectral features are concentrated in the amide III and lipids bands while the band at 1410 cm^−1^ was not reported in studies other than [43]. The weak shoulder at 1685 cm^−1^ is present in two of the studies [15,43] while it was not evident in the third one [37]; this possibly shows that amide I alterations are tumor specific and readily expressed in MM but not in BCC.

Amide III band also exhibited blue-shift and intensity decrease in *in vitro* studies [59], consistent with secondary protein structure changes in BCC. Moreover, it was shown that the CH_2_ bending mode (lipids and protein) was shifted to higher frequencies and the v(PO_2_) symmetric stretching at 1085 cm^−1^ to lower frequency. The bands assigned to proteins, lipids, nucleic acids, and hemoglobin (832, 925, 943, 1006, 1034, 1130, 1209, 1343 and 1607 cm^−1^) were used by Silveira *et al.* [44] for constructing an algorithm for skin cancer diagnosis. Based on Raman spectroscopy determinations of the relative concentrations of core biochemical tissue constituents (melanin, nucleic acid, elastin, ceramide, actin and phenylalanine), they demonstrated an efficient discrimination between healthy skin and pathological skin lesions (BCC and SCC). Statistically significant differences (ANOVA, *p* < 0.05) between normal skin and BCC and normal skin and SCC were found for the relative concentration of melanin, DNA, actin and phenylalanine. Discriminant analysis with the Mahalanobis distance method (identification of outliers) of the melanin *vs.* phenylalanine yielded an overall accuracy of 72.3% when BCC and SCC were considered as a unique disease group by a sensitivity and specificity of 80.9% and 65.0% respectively. In a recent paper [45], the same research group, found that the spectra of the lesions exhibited higher Raman bands intensities compared with normal skin; BCC in the 1300–1700 cm^−1^ range, while SCC and AK in almost the whole spectral range. The difference spectra showed that BCC feature higher lipids concentration while the spectra of SCC suggest higher contribution from both lipids and proteins. Using partial least squares discriminant analysis (PLS-DA) they achieved 89.1% sensitivity and 94.3% specificity for the discrimination of BCC, SCC and AK from normal tissue and benign lesions. The PCA-DA analysis yielded 82.2% accuracy. Spectral vectors showed differences in the Amide III region attributed to pathologic expression of collagen and triolein. The amide III band was also considered as the most significant Raman spectral feature by Philipsen *et al.* [49] for the discrimination of BCC from MM. MM showed the highest intensity increase of the amide III band while BCC the lowest. Other studies however, showed that MM, unlike BCC, exhibit decrease in the intensity of the amide III band [37,60]. Moreover, the amide III ratio which is defined as the protein to lipid ratio for the bands near 1250 and 1300 cm^−1^ (I_amide-III_/I_1290–1330_) [70] was significantly different (*p* = 0.0075) between MM and BCC. No consistent spectral alterations were noticed in the amide I band region between tumor and normal skin. The authors presented two particularly interesting results from the analysis of skin lesions *in vivo*: (i) the clinical diagnostic efficacy of the Raman spectroscopy does not depend on the skin pigmentation and (ii) there is statistically significant (*p* < 0.05) higher intensity in the water band region in BCC and MM compared to normal skin. Based on their classification scheme (Mann–Whitney test), the diagnostic accuracy was MM 93.3% *vs.* normal skin 96.4% and BCC 88.0% *vs.* normal skin 85.5%. On the other hand, Schleusener *et al.* [41] did not notice any increase in the amide III band nor in the phenylalanine band (~1000 cm^−1^), contrary to [44,46]. They also reported increased melanin content and decrease of the amide I band. Contrary to [46], their results supported a significant increase in the lipids content for MM in accordance to [31,64]. Using partial least squares discriminant analysis (PLS-DA), BCC and SCC were discriminated from normal skin with a balanced accuracy of 73% and 85%, respectively. MM and pigmented nevi (PN) discrimination resulted in a balanced accuracy of 91%. Lieber *et al.* [42] reported a 100% sensitivity and 91% specificity in the differentiation between healthy and malignant tissues (BCC and SCC) with a 95% overall classification accuracy using a fiber-coupled portable Raman microscope. They suggested that the core differences in the Raman spectra between normal and malignant lesions mainly rely on divergent protein and lipid compositions of these tissues. Finally, Karagiannis *et al.* [51] developed a novel method where human MM cells were injected in mice. Tumor growth in this animal model was *in vivo* visualized by acoustic microscopy and analyzed by IR and Raman spectroscopy. Significant changes were observed in the 350–2000 cm^−1^ region of the Raman spectra.

## 4. Conclusions

*In vivo* Raman spectroscopy demonstrates significant potential as an emerging clinical diagnostic technique of high sensitivity and specificity for skin cancer screening. Raman spectroscopy requires no sample preparation and features minimal water interference; thus it can be successfully applied to non-invasive *in vivo* evaluation of skin lesions. Results show that real time Raman spectroscopy is able to provide high diagnostic accuracy with acceptable sensitivity and specificity for the effective detection of subtle biochemical alterations in skin tumors with adequate safety and without the need for any tissue pre-treatment. Nevertheless, experimental data show that there are no characteristic Raman peaks that can be uniquely assigned to a particular skin cancer type and significant variations can only be exposed by the application of statistical methods. The problem that multivariate analyses try to solve is two-fold: (i) to expose certain biochemical moieties that mainly contribute to the Raman signals of underline pathologies and (ii) to provide skin cancer screening tests with high sensitivity and specificity for the discrimination between normal and malignant tissues and between different skin lesions. This review showed that the complex environment of the skin tissue provides limited information on the former and complementary techniques may be beneficial. On the contrary, high discrimination accuracy is evident in most cases. The transition from laboratory bench to clinical bed-side setting still remains a challenging task that will take some time to mature. Several prerequisites have to be fulfilled, including optimizing diagnostic accuracy with minimal classification errors, adequate solutions for ethical prerequisites for the *in vivo* use, and fulfillment of all issues that guarantee complete compliance of the portable Raman spectrophotometers with the directives for medical devices. Finally, biopsy with histopathologic assessment, to date the reference procedure for the diagnosis of malignant skin lesions, cannot be replaced by any spectroscopic non-invasive approaches before large-scale comparative clinical trials have established their clinical confidence. Until then, real time, clinical acquisition of Raman spectra can play a supplementary but significant role towards optimizing skin tumor management.
